# Amniotic Fluid Embolism Pathophysiology Suggests the New Diagnostic Armamentarium: β-Tryptase and Complement Fractions C3-C4 Are the Indispensable Working Tools

**DOI:** 10.3390/ijms16036557

**Published:** 2015-03-23

**Authors:** Francesco Paolo Busardò, Paola Frati, Simona Zaami, Vittorio Fineschi

**Affiliations:** 1 Department of Anatomical, Histological, Forensic and Orthopaedic Sciences, Sapienza University of Rome, Viale Regina Elena 336, 00161 Rome, Italy; E-Mails: fra.busardo@libero.it (F.P.B.); simona.zaami@uniroma1.it (S.Z.); 2 Neuromed, Istituto Mediterraneo Neurologico (IRCCS), Via Atinense 18, Pozzilli, 86077 Isernia, Italy; E-Mail: paola.frati@fastwebnet.it

**Keywords:** amniotic fluid embolism, immune response, mast cell degranulation, complement, immunohistochemistry

## Abstract

Amniotic fluid embolism (AFE) is an uncommon obstetric condition involving pregnant women during labor or in the initial stages after delivery. Its incidence is estimated to be around 5.5 cases per 100,000 deliveries. Therefore, this paper investigated the pathophysiological mechanism, which underlies AFE, in order to evaluate the role of immune response in the development of this still enigmatic clinical entity. The following databases (from 1956 to September 2014) Medline, Cochrane Central, Scopus, Web of Science and Science Direct were used, searching the following key words: AFE, pathophysiology, immune/inflammatory response, complement and anaphylaxis. The main key word “AFE” was searched singularly and associated individually to each of the other keywords. Of the 146 sources found, only 19 were considered appropriate for the purpose of this paper. The clinical course is characterized by a rapid onset of symptoms, which include: acute hypotension and/or cardiac arrest, acute hypoxia (with dyspnoea, cyanosis and/or respiratory arrest), coagulopathies (disseminated intravascular coagulation and/or severe hemorrhage), coma and seizures. The pathology still determines a significant morbidity and mortality and potential permanent neurological sequelae for surviving patients. At this moment, numerous aspects involving the pathophysiology and clinical development are still not understood and several hypotheses have been formulated, in particular the possible role of anaphylaxis and complement. Moreover, the detection of serum tryptase and complement components and the evaluation of fetal antigens can explain several aspects of immune response.

## 1. Introduction

Amniotic fluid embolism (AFE) is an uncommon obstetric condition involving pregnant women during labor or in the initial stages after delivery [1,2]. Its incidence is estimated to be around 5.5 cases per 100,000 deliveries [3].

The clinical course is characterized by a rapid onset of symptoms, which include: acute hypotension and/or cardiac arrest, acute hypoxia (with dyspnoea, cyanosis and/or respiratory arrest), coagulopathies (disseminated intravascular coagulation and/or severe hemorrhage), coma and seizures [3,4].

This pathology is burdened with a high rate of mortality and morbidity and possible permanent neurological sequelae for those women who survive [5]. Busardò *et al.* [6] have reported in a recent review a mean mortality rate of 24.8% (±10.96) in affected women, whereas the incidence of AFE in pregnancy related deaths was of 12.8% (±6.5) [6].

Presently, numerous aspects involving the pathophysiology and clinical development are still not understood and several hypotheses have been formulated [7], in particular a possible role of anaphylaxis as hypothesized already in 1956 by Attwood [8] and the role of the complement, which was suggested for the first time in 1982 by Jacob *et al.* [9], in the onset of adult respiratory distress as a consequence of AFE. Therefore, the aim of this paper is to investigate the pathophysiological mechanism that underlies AFE, in order to evaluate the role of the immune response in the development of this still enigmatic clinical entity.

## 2. Methods

The following databases (from 1956 to January 2014) Medline, Cochrane Central, Scopus, Web of Science and Science Direct were used, searching the following key words: Amniotic fluid embolism (AFE), pathophysiology, immune response, complement and anaphylaxis. The main key word “AFE” was searched singularly and then associated individually to each of the other keywords.

**Figure 1:**
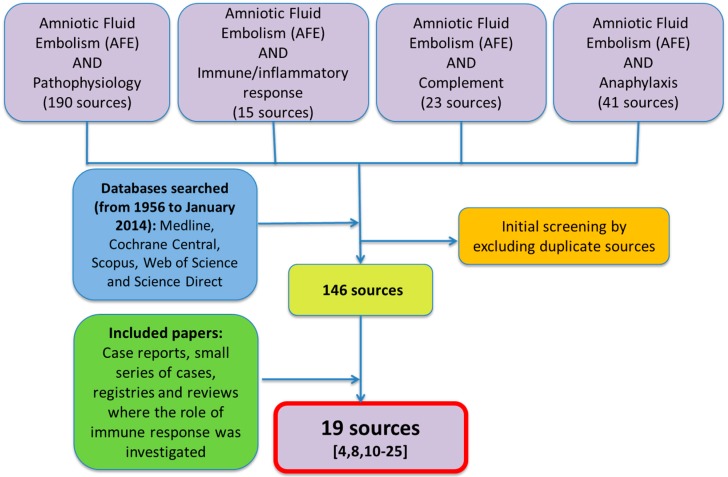
Flow-chart of the papers selected for the purposes of the study.

Of the 146 sources found, only 19 were considered appropriate for the purpose of this paper. All sources have been screened independently by three physicians and in order to be included they had to be selected by at least two of them. A flowchart of the papers selected is reported in Figure 1.

## 3. Results

### 3.1. The Role of β-Tryptase in AFE

Attwood was the first author [8] to hypothesize in 1956 of a possible anaphylactic reaction in the development of AFE in a fatal case involving a 43-year-old woman at her fourth pregnancy. Clark *et al*. [4], several years later in the analysis of the American National registry for AFE, suggested a significant similarity of the clinical course and the hemodynamic and laboratory findings between women with AFE and subjects with anaphylactic shock, in 46 affected patients after the examination of their medical records.

**Figure 2:**
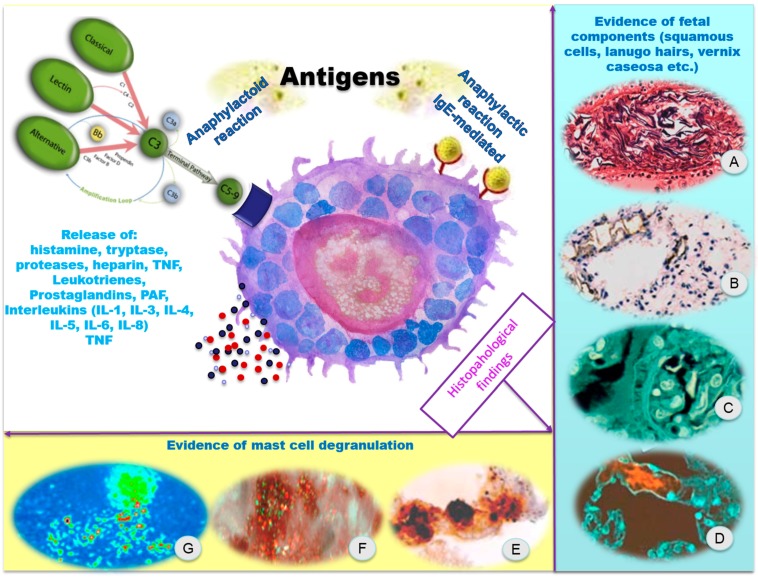
The immuno-inflammatory pathogenesis and the target organs. (**A**) Evidence of fetal squamous cells, lanugo hairs, vernix caseosa, in the pulmonary artery vasculature (Hematoxylin-eosin staining, 200×); (**B**) AE1/AE3 cytokeratin stains on the lungs showed intense intravascular positivity of fetal squamous cells; (**C**) Lanugo hairs and nuclei of fetal squamous cells are clearly visible (confocal laser scanning microscope); (**D**) Mucin fluoresced (in red) in the pulmonary capillary septa (confocal laser scanning microscope); (**E**) Pulmonary capillary septa: evidence of degranulating mast cells with tryptase-positive material outside the cells (Ab anti-tryptase); (**F**,**G**) Degranulating mast cells with tryptase-positive material outside the cells (confocal laser scanning microscope). Figures extracted from cases previously published by Fineschi *et al.* [12].

The anaphylactic reaction as well as septic shock involves the passage into the bloodstream of foreign agents (e.g., bacterial endotoxin, certain antigens, *etc.*), which cause, directly or indirectly, the release of numerous endogenous mediators, responsible for the onset of a typical clinical presentation, including a profound myocardial depression and decreased cardiac output, pulmonary hypertension and disseminated intravascular coagulation (DIC) (Figure 2).

These clinical and hemodynamic disorders are theoretically indistinguishable from those seen in AFE. Moreover, for the first time the term “anaphylactoid” was also introduced, to give a new definition of the disease in “a more descriptive manner as anaphylactoid syndrome of pregnancy” [4]. Anaphylactoid reactions refer to an identical clinical pattern seen in the classical anaphylaxis, non-IgE mediated and certain allergens, including drugs can trigger the mast cell cascade directly without involving IgE as the initial mediator [10].

Mast cells have a key role in inflammatory and immediate allergic reactions. β-tryptase is a serine protease and is the main mediator contained into mast cell granules. The release of β-tryptase from the secretory granules is a typical characteristic of mast cell degranulation. Mast cell β-tryptase plays a significant role in inflammation and acts as a marker of mast cell activation; it activates the protease activated receptor type 2. There is an increase of serum mast cell β-tryptase levels both in anaphylaxis and in other allergic states and is strongly indicative of an immunologically mediated reaction even though it can also happen after a direct activation of mast cells [11].

Regarding the role of mast cell degranulation in the physiopathological mechanism of AFE, different considerations should be placed concerning serological and histological findings. In two different reports [12,13], the Authors detected serum tryptase levels in fatal cases of AFE and the following concentrations were found: 47.2 [12] and 67.2 ng/mL [13].

In comparing both values to the control group of Nishio *et al.* [13], which was composed of 11 cases (three pulmonary embolisms, three traffic accidents, two strangulations, two throttling cases and one anaphylactic shock), the results showed that apart from the case of anaphylactic shock, in which the serum tryptase concentration was 648 ng/mL (17.7 and 9.6 times, respectively, higher than AFE cases), for the remaining 10 controls the mean concentration was of 6.8 ng/mL (range 3.4–9.8 ng/mL), which is 6.9 and 9.9 times, respectively, lower than the two AFE cases. The data above regarding serum tryptase levels seem to support a mast cell degranulation in the physiopathological mechanism of AFE, taking into account the upper normal limits for tryptase concentrations in serum proposed by Edston *et al.* [14] of 44.3 ng/mL. Moreover, according to Mayer *et al.* [15], while moderately elevated tryptase levels are common in post mortem sera, values above 45 ng/mL may support the diagnosis of fatal anaphylaxis.

However, other reports of AFE cases, in which serum tryptase was detected, gave discordant results to the anaphylactic hypothesis.

A 40-year-old woman experienced an acute onset of facial erythema, eclampsia-type seizures, severe hypoxia, cardiac arrest and DIC while in early active labor. The patient died almost 40 min after the onset of symptoms. The autopsy followed by a histological examination showed fetal squames within the pulmonary tree, uterine blood vessels and brain. A peripheral venous blood specimen, obtained approximately one and a half hours postmortem, revealed a tryptase level of 4.7 ng/mL [16]. This level falls within the normal post mortem serum tryptaserange, which does not support the diagnosis.

Benson *et al.* [17], in the analysis of a series of cases involving nine women with “presumed” amniotic fluid embolism and a control group of 22 women who had had a normal labor, detected serum levels of tryptase, urinary histamine concentrations, and serum levels of a fetal antigen; the results obtained were negative for serum tryptase and urinary histamine measurements in women with AFE.

Different considerations should be placed regarding mast cell degranulation in lungs. In a study from 1998 [18], the authors examined the presence and the pulmonary distribution of mast cell tryptase utilizing specific immunohistochemical studies and morphometric evaluation in six fatal cases of AFE, compared to six subjects who died following anaphylactic shock and two control groups (five and six cases, respectively) of traumatic deaths. The results demonstrated a numerical increase of pulmonary mast cells in the subjects who died of AFE (average cell number 54,095) with values corresponding to those encountered in the cases of death due to anaphylactic shock (average cell number 51,378) compared with that of the traumatic control groups (average cell number 24,477 and 9995, respectively). In a more recent paper from 2009 [12], a similar approach was applied and the results obtained showed a large increase in extracellular tryptase consistent with mast cell degranulation in the eight women in the AFE group compared to a control group composed of six pregnant women who died as a result of traumatic injuries. These studies, therefore, highlight that mast cell degranulation occurs in the lungs of AFE fatal cases, whereas it does not occur in other fatal pregnancies.

### 3.2. The Role of Complement in AFE

Hammerschmidt *et al.* [19], in a report from 1984 involving a “30-year-old woman died with massive pulmonary microvascular leukostasis immediately after cesarean hysterectomy”, have postulated the hypothesis of complement activation in the physiopathological mechanism of AFE.

**Figure 3:**
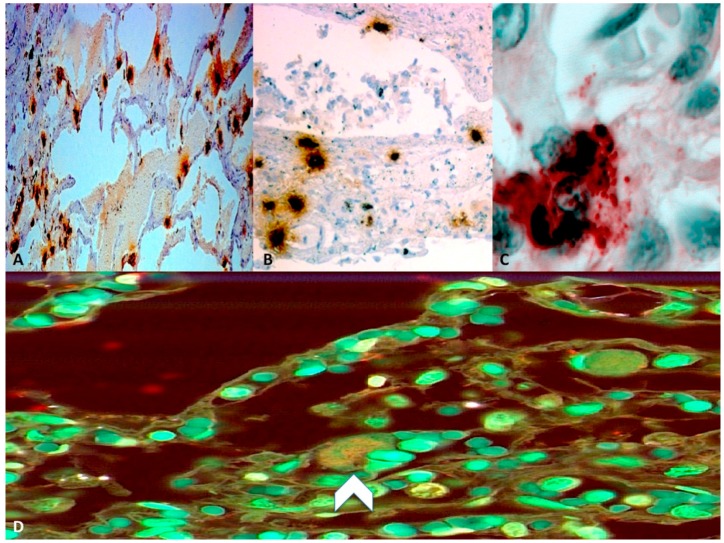
Histopatological findings. (**A**) Pulmonary mast cells with anti-tryptase reactions; (**B**) Degranulating mast cells mast cells present in a bronchial wall (Ab anti-tryptase); (**C**) Evidence of degranulating mastcells with tryptase-positive material outside the cells (Ab anti-tryptase); (**D**) Confocal laser scanning microscope: weak expression (head-arrow) of complement C3a in an AFE case. Figures extracted from cases previously published by Fineschi *et al.* [12].

More recently, Benson *et al.* [17] in the group of eight women with AFE already discussed, detected C3 and C4 levels in serum samples and the results of these analyses showed abnormally low levels of complement. Mean C3 level of 44.0 mg/dL and C4 level of 10.7 mg/dL were significantly lower than corresponding postpartum control values of 117.3 and 29.4 mg/dL. Moreover, postpartum C3 and C4 levels decreased by 8% and 5%, respectively, compared with intrapartum values but they were still within the normal range [17]. In the fatal case of AFE reported by Nishio *et al.* [13], serum C3 and C4 levels showed the following concentrations: 74 and 16 mg/dL, respectively. These values were lower than reported postpartum control values of Benson *et al.* [17] and they are, therefore, significantly suggestive of complement activation. It is important to take into consideration complement levels during normal pregnancies. A study involving ten women in labor found that complement levels remained normal, whereas a more comprehensive view of complement was obtained in two additional groups of normal parturients, in which complement levels were drawn several times during labor and then again immediately post-partum. The results showed that complement components did not undergo a significant change during labor, while there was a statistically significant drop in the minutes after birth [26,27]. Fineschi *et al.* [12], in the analysis of eight fatal cases of AFE, adopted a diagnostic immunohistochemical approach using anti-C3a antibodies and the levels of complement C3a were twofold lower in AFE cases than in the control cases, suggesting a possible complement activation in the physiopathology of the disease (Figure 3). A complement activation reaction to sialyl-Tn (STN) may explain the mechanism of the disease [12]. STN is a known component of meconium and mucin deriving from amniotic fluid. The detection of STN antigen in the serum of patients with suspected AFE constitutes a direct diagnostic method in confirming that the release of amniotic fluid-derived mucin has crossed over into the maternal circulation [20,21]. Complement activation was found along with high levels of STN and levels of complement C3 and C4 were two- to threefold lower than normal [17]. The complement system can be activated by three biochemical pathways: the classical, the alternative and the lectin pathway, which generate homologous variants of the protease C3-convertase; both C3 and C4 are involved in the classical pathway of complement, which normally requires antigen: antibody complexes (immune complexes) for activation. The classical pathway is characterized by the activation of the C1-complex, which then splits C4 and C2 with the formation of C4b2a complex (C3-convertase). The classical pathway C3-convertase cleaves C3 into C3a and C3b, determining a cascade of additional cleavage and activation events. Although the involvement of C3 and C4 is suggestive of the activation of the classical pathway of complement [27], the alternative complement pathway cannot be excluded [19].

In a recent case presented by Hikiji *et al.* [22], involving a 39-year-old woman who died during delivery after a normal 40-week second pregnancy, the authors after a complete post mortem examination, performed an immunohistochemical staining for C5a receptor (C5aR), which was positive in stromal cells around the pulmonary capillaries and inflammatory cells in alveolus. Moreover, they detected concentrations of ZnCP-I and STN significantly higher than the normal values (72.5 pmol/mL and 2630 U/mL, respectively). These findings are consistent with a recent paper by Toro *et al.* [23], who introduced C5aR stain as a helpful technique to prove the complement activation and anaphylatoxin formation and at the same time they support the hypothesis of complement activation initiated by fetal antigens leaking into the maternal circulation [12]. In a recently published study by Tamura *et al.* [24], the authors measured C1 esterase inhibitor (C1INH) activity levels in 106 AFE cases (21 of which were fatal) and the results obtained were compared to a control group composed of 88 women, without AFE and any medical intervention except general birth and surgical assistance. The comparison of the two groups highlighted C1INH activity levels in the AFE cases that were significantly lower than those in the controls (*p* < 0.0001). Moreover, a statistically significant difference (*p* = 0.0121) in C1INH activity levels between fatal (22.5% ± 3.4%) and nonfatal AFE cases (32.0% ± 2.1%) was found.

C1INH represents the major inhibitor of C1 esterase but also of coagulation factor XII activation (FXIIa) and kallikrein [28,29]; therefore, it is able not only to inhibit the complement system but also to modulate the coagulofibrinolytic and kallikrein-kinin systems [30,31]. From a clinical point of view, DIC is considered one of the most common symptoms in AFE patients, and FXII may be involved in its onset because it is activated by several biological and non-biological negatively charged surfaces, determining blood coagulation and the activation of the complement and the kallikrein-kinin system in order to produce bradykinin [32], which has vasodilation effects, a hypotensive effect and causes an increase in vascular permeability resulting in a hypotonic uterus [33,34]. Moreover, FXII is also able to inactivate plasminogen activator inhibitor 1 and enhance fibrinolysis [35]. These evidences are suggestive of the FXII involvement after its activation by contact in the pathophysiological mechanism of AFE. The study of Tamura and colleagues [24], demonstrates that C1INH activity levels in AFE cases were significantly lower than those of controls, especially in fatal cases, suggesting that C1INH levels may be related to the severity of the pathology and may represent a prognostic factor.

## 4. Discussion and Conclusions

Presently, the data available about the significance of serum tryptase is too limited to draw exhaustive conclusions and the absence of suitable animal models do not allow this process. Only by performing a systematic analysis of all cases may we clarify some issues. Moreover, in order to obtain more reliable results in the detection of serum tryptase, the post mortem interval (PMI) should be considered [36,37]. Horn *et al.* [36] analyzed serum levels of tryptase from 57 decedents with varying PMI, who all died of presumed non-anaphylactic causes. Average values of serum tryptase within 10-h increments of PMI were calculated and the results obtained demonstrated significant elevations with increasing PMI and the best results in terms of stability were obtained within five hours of death. Therefore, blood samples in fatal cases of AFE should be collected as soon as possible after death, in order to reduce post mortem variations in tryptase concentrations.

Regarding evidence of mast cell degranulation in the maternal pulmonary vasculature, the results so far achieved, allow us to confirm that mast cell degranulation does take place in the lungs in fatal AFE cases and does not do so in other mortal pregnancy conditions [38]. In this regard, however, other aspects concerning pulmonary mast cell involvement need to be clarified, e.g., if it is a primary mechanism of AFE or a secondary process and if mast cell degranulation can happen as a result of complement activation. Complement activation products C3a and C5a are anaphylatoxic peptides released from the amino terminus of the α-chain of the genetically linked components C3 and C5. They bind to the high affinity Immunoglobulin E receptor (FcεRI) expressed on the cell membrane of mast cells, determining their activation and degranulation [39].

Considering the role of complement in the physiopathology of AFE, several studies agree with its involvement, both directly through the activation of the classical pathway or indirectly by the release of anaphylatoxic peptides, therefore, in the near future with the findings of more studies, it may become a reliable diagnostic marker.

Presently, the diagnosis of AFE is principally based on clinical observation of symptoms; therefore rapid diagnosis and immediate interdisciplinary treatment are essential for a good outcome [40]. For those women, who do not survive, a complete autopsy supported by histological and immunohistochemical analysis is fundamental. Moreover, the detection of serum tryptase and complement components and the evaluation of fetal antigens can explain several aspects of immune response, which are still obscure in the development of this pathology [41].

However, although immunological profiling studies may be the best choice in this difficult process [42], several other biomarkers should be better investigated in order to formulate a prompt diagnosis of AFE. The modern techniques of molecular biology and proteomics could also represent a valid tool in implementing the traditional laboratory tests currently available. The most suitable biomarker should include these features: non-invasive and easy to sample, specific and distinguishable from those naturally present during pregnancy and finally the level of specificity and sensitivity has to be enough to avoid false positive and/or negative results [25]. A list of biomarkers potentially or presently available [26,43,44,45,46,47,48,49,50,51,52,53,54,55,56,57,58,59,60,61] is reported in Table 1.

Advances in the understanding of the physiopathological mechanism of AFE, can help the diagnosis and therefore the treatment. This aim can be achieved by a proper assessment and classification of all cases, in the attempt to clarify numerous pathophysiological aspects in order to set the best standard of care possible in the management of these problematic patients with the objective of reducing the mortality rates and the incidence of permanent damage [61]. If on the one hand growing evidences suggest the activation of the immune system at different levels in the pathophysiology of the disease, on the other one, what remains an open question is “Why don’t all mothers develop an immune response to their fetuses?” Presently, immune tolerance, especially during pregnancy shows many obscure points; indeed, it is proved that the passage of fetal material into the maternal circulation regularly occurs without causing any damage to both mother and fetus [27].

**Table 1:** Biomarkers potentially or presently available for the diagnosis of AFE.

Biomarkers	Applications
Activin A	Maternal serum and amniotic fluid levels of Activin A have been shown to increase with gestational age. The AF-to-MS serum ratio for Activin was 5:1.
Brain natriuretic peptide (BNP)	It is approximately 50-times more concentrated in the AF than it is in the MS. It is usually elevated after any pulmonary event/insult. It is a non-specific marker.
CA125	It is 100 times more concentrated in AF when compared to normal MS. Further investigations are required.
Carcinoembryonic antigen (CEA)	It has been found in AF at concentrations in excess of 200 times those of normal MS, but further investigations are required.
Chromogranin A (CgA)	It is 2.5 times more concentrated in the AF than in MS, but this difference may not be specific since it can fluctuate depending on the trimester of the pregnancy.
Endothelin	It has been found to be significantly elevated in rabbits following the infusion of meconium-stained amniotic fluid.
Fetal cell identification in maternal circulation	Only about 50% of patients with AFE had fetal products detected. The presence of squamous cells in the maternal pulmonary artery circulation is no longer considered pathognomonic.
Insulin-like growth factor binding protein 1 (IGFBP-1)	It is 150 times more concentrated in AF than in MS making it potentially useful.
Interleukin-6, Interleukin-8, Tumor Necrosis Factor-alpha-soluble receptor p55 (sTNFp55)	Significantly higher in AF than in MS, they can be useful markers in diagnosing AFE in the presence of maternal systemic inflammatory response syndrome.
Procollagen type I *N*-terminal propeptide (PINP)	It is approximately 450 times more concentrated in AF than MS, newer methods of assessment of PINP are being developed.
Pro-early placenta insulin-like peptide (Pro-EPIL)	It is 10 times more concentrated in the AF when compared to MS, it is higher in women with pathologic conditions.
Pro-opiomelanocortin (POMC)	It is 10-times more concentrated in the AF than in MS. High concentrations at the feto-maternal interface make it a potential biomarker of AFE.
Prostate-specific antigen (PSA)	The PSA concentration increases with gestational age from 11 to 21 weeks, stabilizes, and then decreases at delivery. This variability makes its use as a biomarker for AFE more difficult.
Sialosyl Tn (STN)	It constitutes a direct diagnostic method of confirming the presence of AF-derived mucin into the maternal circulation. It may be prognostic in maternal mortality.
Squamous cell carcinoma (SCC) antigen	It is known to be released from fetal epidermis and was found in amniotic fluid samples at concentrations over 410 times greater than in maternal serum.
Tissue polypeptide-specific (TPS) antigen	It is 10–20 times more concentrated in AF than in MS Its use as a biomarker for AFE requires extensive additional work.
TKH-2	It represents a more sensitive method of detecting meconium and AF-derived mucin in the lung secretions of patients with AFE, in over 90% of cases.
Zinc Coproporphyrin 1 (ZnCP-1)	It has been found to be significantly elevated in patients with AFE. It is a noninvasive and sensitive method for diagnosing AFE.

AF: Amniotic fluid; MS: Maternal serum; AFE: Amniotic fluid embolism.

Amniotic fluid embolism currently remains not only a clinical nightmare [62], but also an open interdisciplinary challenge [40], which needs the support of obstetricians, intensive care physicians, forensic pathologists and even immunologists.
